# Supplementary material for: Susceptibility to Vaccine-Preventable Diseases in Four Districts of Xaysomboun Province, Lao People’s Democratic Republic
Source: Vaccines (Basel). 2022 Mar 17;10(3):463. doi: 10.3390/vaccines10030463 (PMC8952207; doi:10.3390/vaccines10030463)
Supplement: Supplementary file 1 [file vaccines-10-00463-s001.zip › vaccines-1558784-supplementary.pdf]

Table S1. Factors associated with anti-HBc and HBsAg positivity.

	Anti-HBc					HBsAg				
	**n/N (%)**	**Crude OR [95% CI]**	***p-value***	**Adjusted OR [95% CI]**	***p-value***	**n/N (%)**	**Crude OR [95% CI]**	***p-value***	**Adjusted OR [95% CI]**	***p-value***
Total	204/363 (56.2)					34/363 (9.4)				
**Sex**										
Females	130/222 (58.6)	1.0				17/222 (7.6)	1.0			
Males	74/141 (52.5)	0.78 [0.51-1.19]	0.2	NS	NS	17/141 (12.1)	1.65 [0.81-3.35]	0.1	2.22 [1.03-4.76]	0.04
**Age groups (years)**										
5-10	10/72 (13.9)	1.0				1/72 (1.4)	1.0			
11-20	33/85 (38.8)	3.93 [1.77-8.73]	0.001	3.98 [1.73-9.13]	<0.001	7/85 (8.2)	6.37 [0.76-53.07]	0.08	8.03 [0.94-68.39]	0.056
21-30	48/66 (72.7)	16.53 [6.99-39.06]	<0.001	9.58 [3.04-30.10]	<0.001	7/66 (10.6)	8.42 [1.00-70.42]	0.04	11.98 [1.38-103.87]	0.02
31-40	39/45 (86.7)	40.3 [13.56-119.68]	<0.001	21.33 [5.26-86.53]	<0.001	9/45 (20.0)	17.75 [2.16-145.60]	0.007	30.39 [3.48-264.98]	0.002
> 40	74/95 (77.9)	21.8 [9.57-49.86]	<0.001	9.99 [2.92-34.13]	<0.001	10/95 (10.5)	8.35 [1.04-66.83]	0.04	10.11 [1.23-82.46]	0.03
**Ethnicity**										
Lao-Tai	85/141 (60.3)	1.0				15/141 (10.6)	1.0			
Hmong-Mien	79/156 (50.6)	0.67 [0.42-1.07]	0.09	NS	NS	14/156 (8.9)	0.82 [0.38-1.78]	NS	NA	NA
Mone-Kmae	40/66 (60.6)	1.01 [0.55-1.84]	NS	NS	NS	5/66 (7.6)	0.68 [0.23-1.98]	NS	NA	NA
**Religion**										
Animist	133/243 (57.7)	1.0				20/243 (8.2)	1.0			
Buddhist	71/120 (59.2)	1.19 [0.76-1.86]	NS	NA	NA	14/120 (11.7)	1.47 [0.71-3,02]	0.2	NS	NS
**District**										
Longxarn	44/93 (47.3)	1.0				6/93 (6.4)	1.0			
Hom	48/83 (57.8)	1.52 [0.84-2.77]	0.1	NS	NS	7/83 (8.4)	1.33 [0.43-4.14]	NS	NS	NS
Longchaeng	47/82 (57.3)	1.49 [0.82-2.71]	0.1	NS	NS	5/82 (6.1)	0.94 [0.27-3.20]	NS	NS	NS
Thathom	65/105 (61.9)	1.80 [1.02-3.18]	0.04	3.16 [1.54-6.49]	0.002	16/105 (15.2)	2.60 [0.97-6.97]	0.05	3.15 [1.13-8.79]	0.02
**Place of birth**										
Provincial hospital	3/18 (16.7)	1.0				0/18 (0)	1.0			
District hospital	7/41 (17.1)	1.02 [0.23-4.53]	NS	NS	NS	1/41 (2.4)	0.19 [0.02-1.43]	0.1	NS	NS
Health care center	3/19 (15.8)	0.93 [0.16-5.38]	NS	NS	NS	0/19 (0)				
At home	191/285 (67.0)	10.15 [2.87-35.95]	<0.001	NS	NS	33/285 (11.6)				
**Marital status**										
Single	43/155 (27.7)	1.0				6/155 (3.9)	1.0			
Married	161/208 (77.4)	8.92 [5.52-14.40]	<0.001	NS	NS	28/208 (13.5)	3.86 [1.55-9.57]	0.004	NS	NS
**Education**										
None	47/118 (39.8)	1.0				8/118 (6.8)	1.0			
Elementary	77/123 (62.6)	2.52 [1.50-4.24]	<0.001	NS	NS	12/123 (9.8)	1.48 [0.58-3.77]	NS	NS	NS
Secondary/high school	69/107 (64.5)	2.74 [1.59-4.71]	<0.001	NS	NS	12/107 (11.2)	1.73 [0.68-4.42]	0.1	NS	NS
University	11/15 (73.3)	4.15 [1.24-13.82]	<0.001	NS	NS	2/15 (13.3)	2.11 [0.40-11.04]	NS	NS	NS
**Family income**										

< 1,000,000	22/33 (66.7)					2/33 (6.1)	1.0			
1,000,000 - 3,000,000	164/301 (54.5)	0.59 [0.28-1.27]	0.1	NA	NA	28/301 (9.3)	1.58 [0.36-6.99]	NS	NA	NA
> 3,000,000	18/29 (62.1)	0.81 [0.28-2.32]	NS	NS	NS	4/29 (13.8)	2.48 [0.41-14.66]	NS	NA	NA

Categories were included in multivariable analysis when p≤0.2 for one of the variables.
NS=non-significant, NA=Not appropriate/not included in multivariable analysis.

Table S2. Factors associated with serological profile suggestive of previous vaccination (anti-HBs positive/anti-HBc negative).

	n/N (%)	**Crude OR [95% CI]**	***p-value***	**Adjusted OR [95% CI]**	**p-value**
Total	50/363 (13.8)				
**Sex**					
Female	21/222 (9.5)	1.0			
Male	29/141 (20.6)	2.47 [1.35-4.54]	0.003	2.06 [1.10-3.86]	0.02
**Age groups (years)**					
5-10	21/72 (29.2)	1.0			
11-20	15/85 (17.6)	0.52 [0.24-1.10]	0.09		
21-30	6/66 (9.1)	0.24 [0.09-0.64]	0.005	0.25 [0.09-0.68]	0.007
31-40	0/45 (0)				
> 40	8/95 (8.4)	0.22 [0.09-0.54]	0.001	0.23 [0.09-0.57]	0.001
**Ethnicity**					
Lao-Tai	16/141 (11.3)	1.0			
Hmong-Mien	28/156 (17.9)	1.70 [0.88-3.31]	0.1	NS	NS
Mone-Kmae	6/66 (9.1)	0.78 [0.29-2.09]	NS	NS	NS
**Religion**					
Animist	37/243 (15.2)	1.0			
Buddhist	13/120 (10.8)	0.67 [0.34-1.32]	0.2	NS	NS
**District**					
Longxarn	11/93 (11.8)	1.0			
Hom	16/83 (19.3)	1.78 [0.77-4.09]	0.1	NS	NS
Longchaeng	12/82 (14.6)	1.27 [0.53-3.07]	NS	NS	NS
Thathom	11/105 (10.5)	0.87 [0.35-2.11]	NS	NS	NS
**Place of birth**					
Provincial hospital	7/18 (38.9)	1.0			
District hospital	11/41 (26.8)	0.57 [0.17-1.86]	NS	NS	NS
Health care center	5/19 (26.3)	0.56 [0.13-2.26]	NS	NS	NS
At home	27/285 (9.5)	0.16 [0.05-0.45]	0.001	NS	NS
**Marital status**					
Single	37/155 (23.9)	1.0			
Married	13/208 (6.2)	0.21 [0.10-0.41]	<0.001	NS	NS
**Education**					
None	26/118 (22.0)	1.0			
Elementary	10/123 (8.1)	0.31 [0.14-0.68]	0.004	NS	NS
Secondary/high school	10/107 (9.3)	0.36 [0.16-0.79]	0.01	NS	NS
University	4/15 (26.7)	1.28 [0.37-4.37]	NS	NS	NS
**Family income**					
< 1,000,000	3/33 (9.1)	1.0			
1,000,000 - 3,000,000	44/301 (14.6)	1.71 [0.50-5.85]	NS	NA	NA
> 3,000,000	3/29 (10.3)	1.15 [0.21-6.21]	NS	NA	NA

Categories were included in multivariable analysis when p≤0.2 for one of the variables. NS=non-significant, NA=Not appropriate/not included in multivariable analysis

Table S3. Factors associated with anti-measles seropositivity and with anti-rubella seropositivity.

	**Anti-measles seropositivity**					**Anti-rubella seropositivity**			
	**n/N (%)**	**Crude OR [95% CI]**	**p-value**	**Adjusted OR [95%CI]**	**p-value**	**n/N (%)**	**Crude OR [95% CI]**	**p-value**	**Adjusted OR [95% CI]**
**Total**	220/363 (60.6)					331/363 (91.2)			
**Sex**									
Female	149/222 (67.1)	1.0				201/222 (90.5)	1.0		
Male	70/141 (50.3)	0.49 [0.32-0.76]	0.002	0.58 [0.33-1.00]	NS	130/141 (92.2)	1.23 [0.57-2.64]	NS	NA
**Age groups (years)**									
5-10	18/72 (25.0)	1.0				66/72 (91.7)	1.0		
11-20	27/85 (31.8)	1.39 [6.69-2.81]	NS	NS	NS	83/85 (97.6)	3.77 [0.73-19.30]	0.1	NS
21-30	48/66 (72.7)	8.00 [3.74-17.11]	<0.001	3.47 [1.20-10.06]	0.02	57/66 (86.3)	0.57 [0.19-1.71]	NS	NS
31-40	41/45 (91.1)	30.75 [9.66-97.79]	<0.001	10.44 [2.45-44.42]	0.001	41/45 (91.1)	0.93 [0.24-3.50]	NS	NS
> 40	86/95 (90.5)	28.66 [12.01-68.39]	<0.001	10.48 [2.97-37.02]	< 0.001	84/95 (88.4)	0.69 [0.24-1.97]	NS	NS
**Ethnicity**									
Lao-Tai	88/141 (62.4)	1.0				129/141 (91.5)	1.0		
Hmong-Mien	94/156 (60.3)	0.91 [0.57-1.45]	NS	NA	NA	142/156 (91.0)	0.94 [0.42-2.11]	NS	NA
Mone-Kmae	38/66 (57.6)	0.81 [0.45-1.48]	NS	NA	NA	60/66 (90.9)	0.93 [0.33-2.59]	NS	NA
**Religion**									
Animist	145/243 (59.7)	1.0				223/243 (91.8)	1.0		
Buddhist	75/120 (62.5)	1.12 [1.14-1.19]	NS	NA	NA	108/120 (90.0)	0.80 [0.38-1.71]	NS	NA
**District**									
Longxarn	56/93 (60.2)	1.0				81/93 (87.1)	1.0		
Hom	58/83 (69.9)	1.53 [0.81-2.86]	0.1	NS	NS	79/83 (95.2)	2.92 [0.90-9.45]	0.07	NS
Longchaeng	49/82 (59.8)	0.98 [0.53-1.79]	NS	NS	NS	77/82 (93.9)	2.28 [0.76-6.77]	0.1	NS
Thathom	57/105 (54.3)	0.78 [0.44-1.38]	NS	NS	NS	94/105 (89.5)	1.26 [0.53-3.02]	NS	NS
**Place of birth**									
Provincial hospital	6/18 (33.3)	1.0				17/18 (94.4)	1.0		
District hospital	9/41 (21.9)	0.56 [0.16-1.92]	NS	NS	NS	39/41 (95.1)	1.14 [0.09-13.52]	NS	NA
Health care center	5/19(26.3)	0.71 [0.17-2.94]	NS	NS	NS	18/19 (94.7)	1.05 [0.06-18.30]	NS	NA

At home	200/285 (70.2)	4.70 [1.71-12.9]	0.003	NS	NS	257/285 (90.2)	[0.06-4.21]	NS	NA
**Marital status**									
Single	44/155 (22.4)	1.0				147/155 (94.8)	1.0		
Married	176/208 (84.6)	13.87 [8.30-23.18]	<0.001	2.7 [1.09-37.02]	0.03	184/208 (88.5)	0.41 [0.18-0.95]	0.03	NS
**Education**									
None	91/118 (51.7)	1.0				107/118 (90.7)	1.0		
Elementary	83/123 (67.5)	1.93 [1.15-3.26]	0.01	NS	NS	109/123 (88.6)	0.80 [0.34-1.84]	NS	NA
Secondary/high school	66/107 (61.7)	1.50 [0.88-2.55]	0.1	NS	NS	100/107 (93.5)	1.46 [0.54-3.93]	NS	NA
University	10/15 (66.7)	1.86 [0.60-5.80]	0.2	NS	NS	15/15 (100)			
**Family income**									
< 1,000,000	19/33 (57.6)	0.1				32/33 (96.9)	1.0		
1,000,000 – 3,000,000	186/301 (61.8)	1.19 [0.57-2.46]	NS	NA	NA	272/301 (90.4)	0.29 [0.03-2.22]	0.2	NS
> 3,000,000	15/29 (51.7)	0.78 [0.28-2.15]	NS	NA	NA	27/29 (93.1)	0.4 [0.03-4.91]	NS	NS

Categories were included in multivariable analysis when p≤0.2 for one of the variables. NS=non-significant, NA=Not appropriate/not included in multivariable analysis

Table S4. Factors associated with anti-tetanus seroprotection (> 0.5 IU/mL).

	n/N (%)	**Crude OR [95% CI]**	*p-value*	**Adjusted OR [95% CI]**	*p-value*
Total	172/363 (47.4)				
**Sex**					
Female	143/222 (64.1)	1.0			
Male	29/141 (20.6)	0.14 [0.08-0.23]	<0.001	0.12 [0.07-0.21]	<0.001
**Age groups (years)**					
5-10	23/72 (31.9)	1.0			
11-20	39/85 (45.9)	1.80 [0.93-3.47]	0.07	2.10 [0.99-4.43]	0.05
21-30	35/66 (53.0)	2.40 [1.20-4.80]	0.01	2.65 [1.17-6.00]	0.01
31-40	34/45 (75.6)	6.58 [2.83-15.27]	<0.001	6.47 [2.48-16.85]	<0.001
> 40	41/95 (43.2)	1.61 [0.85-3.06]	<0.001		
**Ethnicity**					
Hmong-Mien	58/156 (37.2)	1.0			
Mone-Kkmer	32/66 (48.5)	1.59 [0.88-2.84]	0.1	NS	NS
Lao-Tai	82/141 (58.2)	2.34 [1.47-3.74]	<0.001	NS	NS
**Religion**					
Animist	98/243 (40.3)	1.0			
Buddhist	74/120 (61.7)	2.38 [1.52-3.72]	<0.001	2.80 [1.40-5.67]	0.003
**District**					
Longxarn	34/93 (36.6)	1.0			
Hom	40/83 (48.2)	1.61 [0.88-2.94]	0.1	2.37 [1.16-4.86]	0.01
Longchaeng	36/82 (43.9)	1.35 [0.74-2.49]	NS	NS	NS
Thathom	62/105 (59.1)	2.50 [1.40-4.44]	0.002	2.60 [1.19-5.65]	0.01
**Place of birth**					
Provincial hospital	8/18 (44.4)	1.0			
District hospital	16/41 (39.0)	0.80 [0.26-2.45]	NS	NA	NA
Health care center	8/19 (42.1)	0.90 [0.24-3.34]	NS	NA	NA
At home	140/285(49.1)	1.20 [0.46-3.14]	NS	NA	NA
**Marital status**					
Married	61/155 (39.4)	1.0			
Single	111/208 (53.4)	1.76 [1.15-2.68]	0.008	NS	NS
**Education**					
None	45/118 (38.1)	1.0			
Elementary	72/123 (58.5)	2.29 [1.36-3.83]	0.002	NS	NS
Secondary/high school	49/107 (45.8)	1.37 [0.80-2.33]	0.2	NS	NS
University	6/15 (40.0)	1.08 [0.36-3.24]	NS	NS	NS
**Family income**					
< 1,000,000	19/33 (57.6)	1.0			
1,000,000 – 3,000,000	138/301 (45.8)	0.62 [0.30-1.29]	0.2	NS	NS
> 3,000,000	15/29 (51.7)	0.78 [0.28-2.15]	NS	NS	NS

Categories were included in multivariable analysis when p≤0.2 for one of the variables.

NS=non-significant, NA=Not appropriate/not included in multivariable analysis
